# Mediterranean Diet as a Supportive Intervention in Cancer Patients: Current Evidence and Future Directions

**DOI:** 10.3390/curroncol29100597

**Published:** 2022-10-11

**Authors:** Roberta Rubino, Michela Rosaria Iuliucci, Simona Gatani, Arianna Piscosquito, Bruno D’Ambrosio, Concetta Ingenito, Luca Scafuri, Carlo Buonerba, Giuseppe Di Lorenzo

**Affiliations:** 1 Oncology Unit, “Andrea Tortora” Hospital, ASL Salerno, 84016 Pagani, Italy; 2 Associazione O.R.A.—Oncology Research Assistance, 80049 Somma Vesuviana, Italy; 3 Department of Medicine and Health Sciences “Vincenzo Tiberio”, University of Molise, 86100 Campobasso, Italy

Cancer currently represents a leading cause of morbidity and mortality, and it can be held responsible for about one in six deaths worldwide [1]. Nutrition represents a rather novel, promising, highly cost-effective intervention paradigm in oncology, not only because 30–50% of all cancer cases can be prevented with proper diet and lifestyle modifications [2], but also because diet may affect the risk of cancer recurrence [3] and even influence the course of clinically relevant, cancer-related [4] and treatment-related adverse events [5]. Among several nutritional approaches [6] with potential benefits in cancer patients, which include the use of hypocaloric, fasting-mimicking, ketogenic, low-fat diets, the adoption of the Mediterranean Diet (MedDiet) represents one of the most promising, easily applicable and widely investigated nutritional interventions [7]. The characteristics of the MedDiet include olive oil as the main source of fat; daily consumption of fresh fruits, nuts, non-refined cereals, vegetables, low-fat dairy products; low to moderate consumption of eggs, potatoes, ethanol (especially red wine) and poultry; and a low consumption of red meat [8]. The MedDiet may represent a powerful resource in oncology and be applied in several different settings. First, a number of studies support the role of the MedDiet in reducing cancer incidence. In fact, among 605 survivors of a first episode of acute myocardial infarction randomized to follow either the MedDiet or a control diet, cancer rates per 100 patients per year were 0.72 vs. 1.61 in the MedDiet vs. the control group, respectively, after a mean follow-up of 46.7 and 44.9 months, respectively. Reductions of 56% (*p* = 0.03), 61% (*p* = 0.05) and 56% (*p* = 0.01) for total deaths, cancers, and the combination of deaths and cancers, was, respectively, reported in the MedDiet vs. the control group after adjusting for several confounding factors [3]. The ongoing randomized-controlled DIANA-5 trial [9] was designed to test the MedDiet as a tertiary prevention intervention in patients receiving radical treatment for breast cancer. In the cohort of 1344 women who completed the first year of study intervention, those on the MedDiet showed improved metabolic parameters, although a longer follow-up is required to gather conclusive evidence on the influence of the MedDiet on breast cancer recurrence risk [10]. Further evidence is provided by an analysis of the PREDIMED trial that randomized 4282 women aged 60 to 80 years to the MedDiet supplemented with extra-virgin olive oil, MedDiet supplemented with mixed nuts, or a control diet. After a median follow-up of 4.8 years, patients randomized to MedDiet supplemented with extra-virgin olive oil and those randomized to MedDiet supplemented with nuts showed favorable hazard ratios for breast cancer occurrence of 0.32 (95% CI, 0.13–0.79) and of 0.59 (95% CI, 0.26–1.35), compared to the control group, respectively [11]. Additionally, in a cohort of 165 women who had a primary diagnosis of colorectal adenoma and were assessed for dietary habits at the time of diagnosis, a high consumption of olive oil, vegetables, fruit, fish and lean meat significantly reduced adenoma recurrence, although such an association was not found in men [12]. In a comprehensive study of published data obtained in more than 12 million individuals, convincing or highly suggestive evidence supporting the protective effect of adherence to the Mediterranean diet was found for overall cancer incidence and mortality and, among individual tumors, for breast cancer only [8], which may be explained by the association of the MedDiet with lower estrogen levels [13]. Little evidence is available regarding the effects of the MedDiet in patients with active cancer. In a small, prospective trial including 30 patients with advanced small cell lung cancer randomized to a personalized the MedDiet vs. a control diet, the MedDiet was associated with lower levels of inflammatory markers after 3 months, which suggests that it may positively influence cancer prognosis [14]. Another small, randomized trial concluded that a nutritional intervention based on extra-virgin olive oil rich in two polyphenols showing in vitro anti-proliferative properties, oleocanthal and oleacein, could slow the progression of chronic lymphatic leukemia [15]. 

Besides preventing cancer occurrence and recurrence and, potentially, improving cancer-related prognosis, the MedDiet may reduce the risk of specific cancer- and treatment-related adverse events, such as venous thromboembolism [16,17], hypertension, fatigue. The analysis of fruit and vegetable consumption distribution in a prospective cohort of 14,962 middle-aged participants showed that those in the greatest vs. the lowest quintile presented a significant 40% reduction of venous thromboembolism incidence. Additionally, those who ate fish, another major component of the MedDiet, at least once a week showed a significant 30–45% lower incidence of venous thromboembolism, while opposite associations were found for red and processed meat intake [18]. Importantly, preliminary evidence suggests that isoquercetin [19], a flavonol [20] present in tomatoes, capers and most fruit and vegetables, may reduce the incidence of venous thromboembolism in cancer patients, besides improving cancer-related fatigue [21,22]. Lastly, the MedDiet may reduce the incidence of hypertension [23], an adverse event of several anti-cancer drugs [24].

In conclusion, the MedDiet represents a historical, cultural, and gastronomic heritage of humanity. Research on its application in oncology remains in its infancy. Barriers to its broader investigation as an intervention to improve cancer outcomes or decrease cancer-related adverse events in cancer patients or in individuals without cancer risk include difficulties in protecting and monetizing intellectual property and in reproducing the nutritional intervention. Furthermore, it must be considered that most oncology units in Western countries are unable to offer a consultation with a nutritionist. Despite these hurdles, the potential of MedDiet as a supportive measure for cancer patients requires the medical and scientific community to take appropriate actions to continue investigating it and expand its spectrum of applications in oncology. 
